# A mathematical model of aging-related and cortisol induced hippocampal dysfunction

**DOI:** 10.1186/1471-2202-10-26

**Published:** 2009-03-25

**Authors:** Mark T McAuley, Rose Anne Kenny, Thomas BL Kirkwood, Darren J Wilkinson, Janette JL Jones, Veronica M Miller

**Affiliations:** 1 Henry Wellcome Building, Biogerontology Building, Institute for Ageing and Health, Newcastle University, Newcastle Upon Tyne, England, NE4 6BE, UK; 2 Trinity College Institute for Neuroscience, Trinity College, College Green, Dublin 2, Eire; 3 School of Mathematics & Statistics, Newcastle University, Newcastle upon Tyne, NE1 7RU, UK; 4 Unilever R&D, Port Sunlight, Quarry Road East, Bebington, Wirral, England, CH63 3JW, UK; 5 Neurovascular Research Unit, Institute for Ageing and Health, Newcastle General Hospital, Newcastle Upon Tyne, England, NE46BE, UK; 6 Wadsworth Center For Laboratories and Research, NYS Department of Health, PO Box 509, Albany, NY 12201-0509, USA

## Abstract

**Background:**

The hippocampus is essential for declarative memory synthesis and is a core pathological substrate for Alzheimer's disease (AD), the most common aging-related dementing disease. Acute increases in plasma cortisol are associated with transient hippocampal inhibition and retrograde amnesia, while chronic cortisol elevation is associated with hippocampal atrophy. Thus, cortisol levels could be monitored and managed in older people, to decrease their risk of AD type hippocampal dysfunction. We generated an in silicomodel of the chronic effects of elevated plasma cortisol on hippocampal activity and atrophy, using the systems biology mark-up language (SBML). We further challenged the model with biologically based interventions to ascertain if cortisol associated hippocampal dysfunction could be abrogated.

**Results:**

The in silicoSBML model reflected the in vivoaging of the hippocampus and increased plasma cortisol and negative feedback to the hypothalamic pituitary axis. Aging induced a 12% decrease in hippocampus activity (HA), increased to 30% by acute and 40% by chronic elevations in cortisol. The biological intervention attenuated the cortisol associated decrease in HA by 2% in the acute cortisol simulation and by 8% in the chronic simulation.

**Conclusion:**

Both acute and chronic elevations in cortisol secretion increased aging-associated hippocampal atrophy and a loss of HA in the model. We suggest that this first SMBL model, in tandem with in vitroand in vivostudies, may provide a backbone to further frame computational cortisol and brain aging models, which may help predict aging-related brain changes in vulnerable older people.

## Background

Aging-related neurodegenerative diseases are ever increasing thanks to global demographic changes. Alzheimer's disease (AD) is the most common aging-related neurodegenerative disease; the incidence of which doubles yearly after one's seventh decade. Key symptoms of AD are the loss of declarative memory and decreased cognition which are associated with amyloid plaques and tau neurofibrillary tangle deposition and the depletion of hippocampal neurons [1,2]. The loss of hippocampal CA1 neurons is particularly associated with hippocampal atrophy and memory deficits in AD [3]. As yet, the exact pathological mechanism underscoring hippocampal degeneration in AD remains elusive; however aging-related factors such as diabetes, vascular disease and stress (elevated cortisol levels) are common risk factors for AD [4-6]. In the elderly, elevated plasma cortisol levels are associated with hippocampal atrophy, suggesting cortisol is involved in hippocampal dysfunction. Furthermore, the negative effects of elevated plasma cortisol levels on cognition in the elderly can be abrogated by blocking plasma cortisol release [7,8]. This suggests that better plasma cortisol regulation may yield improved hippocampal activity in older people.

Plasma cortisol levels are regulated by the hypothalamic-pituitary-adrenal (HPA) axis and the hippocampus, which interact to form a negative feedback circuit to regulate cortisol release. Cortisol's effect on the hippocampus is mediated through interactions with mineralocorticoid receptors (MR) which increase the firing rate of CA1 neurons. A rise in cortisol levels sufficient to fully saturate MR receptors induces the transcription of inhibitory glucocorticoid receptors (GR) which decrease CA1 neuronal firing in tandem with augmenting negative feedback to the HPA to decrease cortisol secretion [9].

Cortisol provides tonic activation of hippocampal neurons, which can be useful during stressful situations which require alertness and increased neuronal activity. However, a short term overshoot of normal cortisol plasma levels may induce GR transcription, the inhibition of CA1 neuronal activity, and transient amnesia; the "tip of the tongue" phenomenon some experience during stressful situations. Plasma cortisol thus has a "U" shaped dose dependent effect on the firing potential of hippocampal neurons [10]. In the long term elevated cortisol levels are associated with hippocampal atrophy [11-13]. This has led to suggestions that the chronic pathological effects of elevated cortisol levels may be treated in older people by physiological or pharmacological interventions. For example, plasma cortisol levels can be decreased by simple activities of daily living such as exercise [14].

**Figure 1:**
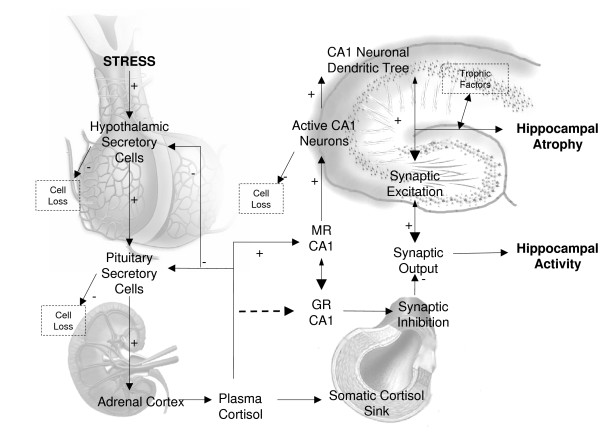
**Physiological Basis of SBML Model**. This figure describes the relationships between cortisol synthesis by the HPA axis and cortisol binding to hippocampal MR receptors with respect to synaptic firing at CA1 hippocampal neurons. Hippocampal atrophy is related to the numbers of neurons within the CA1 region, further defined by the branching of neurons therein. Ageing-related changes are shown chiefly as cell loss from hypothalamic hippocampal and pituitary tissues, along with a decline in the synthesis and availability of trophic factors. Stress associated changes are shown by the dashed arrow to GR receptors whereby elevated cortisol induces the expression of GR receptors, which are associated with CA1 neuronal synaptic inhibition. The combined effects of aging and stress may decrease hippocampal activity- defined as a combination of synaptic excitation and inhibition, and increase hippocampal atrophy.

**Figure 2:**
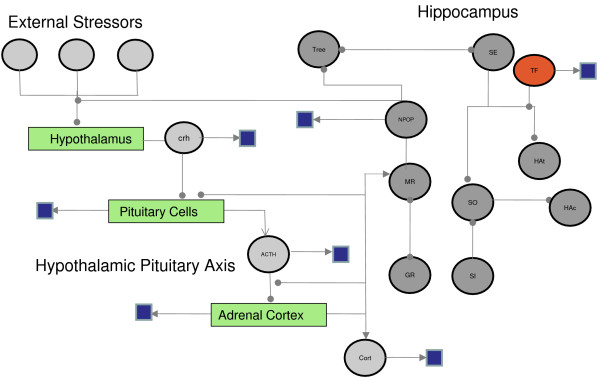
**Network diagram of SBML Model**. This flow chart illustrates the relationship between the main components of the SMBL model and is an abstracted simplified version of the physiological systems (HPA axis, hippocampus etc) modelled.

Physiological studies on the effects of cortisol on hippocampal function are in abundance, as are computational models of HPA regulation of cortisol synthesis. Yet, despite the growing evidence that cortisol is associated with hippocampal dysfunction, there are no in silicomodels of aging-related effects of cortisol on hippocampal function. The benefit of in silicomodelling lies not only in helping to further define and clarify biological systems, but also in providing a medium through which hypotheses which would be difficult and costly to test in vivo*- *a particular feature of aging related studies- can be approached. Therefore, to help define the relationship between plasma cortisol, aging and hippocampal function, we used clinical data on HPA function, plasma cortisol levels and hippocampal activity to generate an in silicomodel using the Systems Biology Markup Language (SBML) (Figure 1 and 2).

## Results

### Effects of chronic and acute changes in cortisol levels on Hippocampal Activity and Atrophy

**Figure 3:**
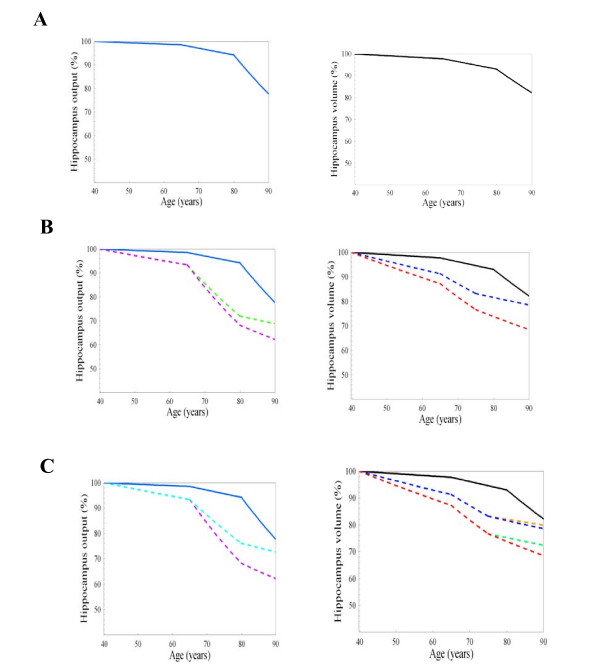
**Simulation of effects of age, acute, chronic and a biological intervention based change in cortisol levels on Hippocampal Activity and Volume**. Graphs produced using MathSBML show the relationship between cortisol and hippocampal activity/volume with respect to a) age b) an acute (blue line) and chronic (dashed line) increase in cortisol and c) acute and chronic stress after an intervention, modelled using parameters described in the methods section using SBML.

The model was simulated under normal aging conditions defined as; a loss of neurons, decreased dendritic arborisation, decreased synaptic density in the hippocampus, a loss of secretory cells in the HPA, altered negative feedback from the hippocampus, and a gradual decrease in the circulating plasma levels of cortisol. These aging conditions were set to represent the normal aging processes in the brain of an individual between the ages of 45–90 years (Figure 3, graph A). A simulation of the effects of acute bursts in plasma cortisol levels induced a decrease in hippocampal output (HO) of 30% in tandem with a 19% decrease in hippocampal volume (HV). A chronic increase in cortisol produced a decrease of 40% in HO associated with a 25% decrease in HV (Figure 3, graph B). The application of a biological intervention to decrease cortisol levels resulted in a 7% abrogation of the acute cortisol induced decrease in HO while the intervention abrogated the chronic cortisol decrease in HA by 22% (Figure 3, graph C). This suggests that a biological intervention may be most effective in relation to a decrease in hippocampal atrophy associated with a chronic elevation in cortisol.

### B) Sensitivity of plasma cortisol to negative feedback at Hypothalamus, Pituitary and CRH levels

**Figure 4:**
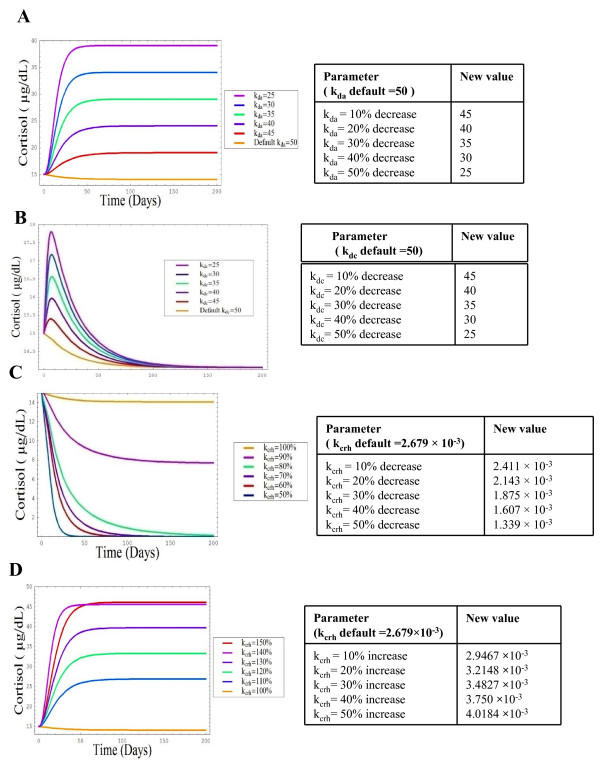
**Response of the model to negative feedback of cortisol regulation from a) the hypothalamus and b) pituitary c) increased CRH and d) decreased CRH production**. Parameters were changed by a factor of 10–50% inducing changes in circulating cortisol levels as described in accompanying tables, modelled using SBML as detailed in the methods section.

There is an aging-related impairment in the feedback inhibition of cortisol on HPA activity [15,16]. Therefore, to test the reflexivity and sensitivity of the model to physiologically based changes in cortisol regulation, challenges to negative feedback at the hypothalamus and pituitary were simulated. Negative feedback at the hypothalamus and the pituitary was decreased between 10–50%. A decrease in the rate of feedback at the hypothalamus induced a gradual increase in plasma cortisol in tandem with a drop in k_da_, the dissociation constant for cortisol at the hypothalmus (Figure 4, graph A). This rise was not continuous; plasma cortisol entered a new steady state almost instantaneously in response to the parameter changes. A maximum basal concentration of 38 ug/dL was reached in response to a 50% increase in k_da_.

The effect of a change in the rate of negative feedback at the level of the pituitary was examined by decreasing k_dc _in the range of 10–50%. An alteration in cortisol feedback at the pituitary produced a rise in plasma cortisol in tandem with each increase in k_dc_, followed by a decrease in plasma cortisol until levels reached a steady state akin to that observed with the default value (Figure 4, graph B).

The next parameter investigated was k_crh_,; the rate constant for the secretion of CRH. k_crh _was decreased in the range 10–50%, each of which produced significant decreases in basal plasma cortisol levels (Figure 4, graph C). When k_crh _decreased by 30, 40 and 50%, the basal plasma cortisol level eventually dropped to zero, reflecting the key role CRH has in the synthesis of cortisol. Conversely when k_crh _was increased in the range of 10–50%, gradual increases in basal plasma cortisol levels were observed. Again plasma cortisol levels reached a steady state instantaneously with each parameter change, but plasma cortisol did not continue to rise further after k_crh _was increased by 40% (Figure 4, graph D).

Altogether these results suggest the in silicosystem responds to negative feedback in a similar fashion to that reported in biological in vivosystems; *ie *the hypothalamus, pituitary and CRH hormone production were all reflexive to negative feedback via sensitivity to plasma cortisol levels.

### C) Response of the system to decreased negative feedback from the hippocampus

**Figure 5:**
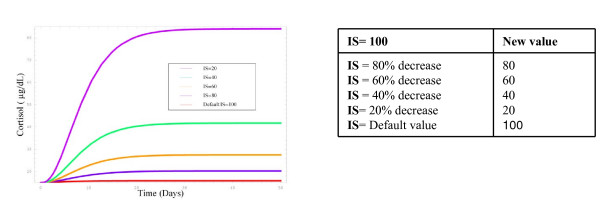
**Response of cortisol levels to changes in negative feedback from the hippocampus**. Parameters were changed by a factor of 20–80%, introducing significant changes in circulating cortisol levels.

The hippocampus provides tonic negative inhibition to the HPA axis, thus when hippocampal output activity levels are high, so too is the degree of negative inhibition from it to the HPA. When hippocampal activity declines due to increased density of inhibitory synapses, compounded by age, then the degree of negative feedback from the hippocampus to the HPA is lessened, thus an increase in cortisol synthesis is seen. Figure 5 shows the relationship between hippocampal activity and HPA regulation of cortisol levels.

### D) Sensitivity of HPA axis to altered cortisol synthesis

**Figure 6:**
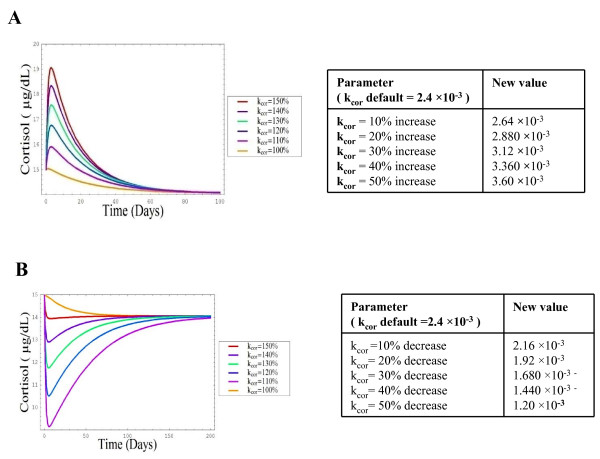
**Simulation showing the effects of a) increased rate of cortisol production and b) decreased rate of cortisol production on plasma cortisol levels**. Parameters were changed by a factor of 10–50% inducing changes in circulating cortisol levels as described in accompanying tables, modelled using SBML as detailed in the methods section.

A number of studies have suggested cortisol secretion alters with age [2,17]. The analysis of the effects altered cortisol synthesis commenced by changing k_cor _(the rate constant for the production of cortisol) in the range 10–50% producing an increase in basal cortisol levels with each parameter increment, with levels reaching a maximum value of 19 ug/dL with the largest parameter alteration. Over time basal plasma cortisol levels returned to the original default value (Figure 6).

These observations suggest that the mechanisms in place to deal with elevations in plasma cortisol (*e.g*. feedback at the level of the pituitary and hypothalamus) ensured that plasma cortisol did not stay elevated for long, thus showing that the simple in silicosystem reflects the biological HPA regulation of cortisol in vivo.

### E) Response of System to Somatic Cortisol Demands

**Figure 7:**
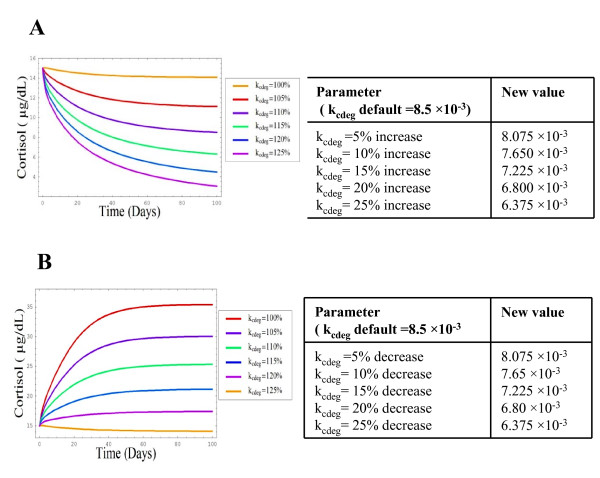
**Plasma Cortisol responses to central cortisol secretion**. Simulations shows the effects of a) increased and b) decreased somatic tissue utilisation of cortisol on plasma cortisol levels. Simulation of the response of the model to parameters were changed by a factor of 10–50% inducing changes in circulating cortisol levels as described in accompanying tables, modelled using SBML as detailed in the methods section.

The final parameter examined was k_cdeg_, the rate constant defining the use of freely available plasma cortisol by somatic tissue. This analysis commenced with an increase of k_cdeg _in the range of 5–25% which produced a progressive decline in plasma cortisol with each parameter increase, followed by an eventual return to steady state levels (Figure 7, graph A). Conversely, a decrease in the rate of cortisol utilisation by somatic tissue (k_cdeg_) produced a progressive rise in cortisol. This increase in cortisol continued until a new steady state was reached (Figure 7, graph B), thus illustrating the sensitivity of the system to plasma cortisol usage.

### F) Parameter changes to examine daily cortisol rhythm

**Figure 8:**
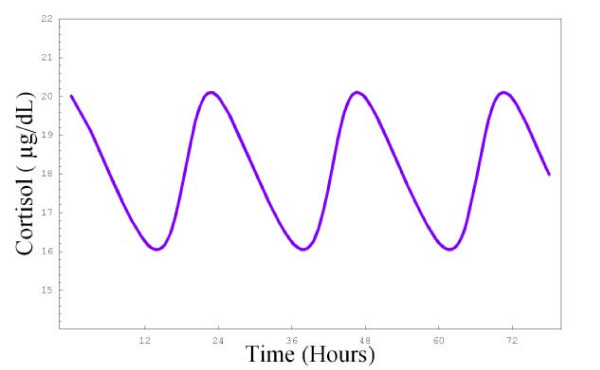
**Diurnal rhythm of cortisol in response to changes in ODEs 1–3 Diurnal oscillations of cortisol over a 72 hour period**. This graph was produced by making changes to equations 1–3. These changes are detailed in the appendix.

We introduced diurnal oscillations in cortisol secretion so as to help modelers wishing to study this aspect of the model. We have outlined the changes to the ODEs in the Appendix that are needed to produce the diurnal rhythms and have given an example of how the diurnal rhythm changes with age (Figure 8).

## Discussion

The physiological mechanisms underpinning the association between elevated plasma cortisol and hippocampal atrophy in the elderly are not fully understood. However, it is accepted that the expression and activation of cortisol receptors influences hippocampal neuronal activity, and that elevated plasma cortisol is associated with hippocampal dysfunction and memory loss [10,18]. The neurotoxic effects of elevated cortisol on the hippocampus most likely involves "allostatic" loading of hippocampal cortisol receptors, accelerated by an aging-related loss of neurons and trophic factors [12,13]. To help further understand the association between stress and hippocampal function, we used information on aging, the HPA axis, plasma cortisol and hippocampal activity, to simulate the effect of chronic and acute cortisol elevations on the hippocampus.

We recognise that cortisol receptors are present throughout the brain, in limbic, brainstem, and cortical regions, all of which are sensitive to aging-related dysfunction. For example, prefrontal cortical (PFC) neurons contribute to cognitive function, particularly executive function; a decrease in which is found in patients with advanced AD, Parkinson's disease and vascular dementia [19,20]. Rodent studies have also demonstrated dystrophy of PFC neurons after exposure to elevated cortisol levels [21]. This suggests that the PFC may be an interesting component to add to the current model in other to expand its utility in terms of understanding the relationship between cortisol and cognitive function. However in this first paper, we wished to model the clinically documented, but ill-understood link between elevated plasma cortisol levels and AD type hippocampal dysfunction. Therefore we focused on the atrophy of the hippocampus and activity of CA1 neurons therein, because the hippocampus regulates semantic memory and verbal fluency- cognitive domains which are lost at early stages of AD.

The simulations produced by the model suggest that chronic elevations in cortisol are more detrimental to the hippocampus than a series of acute bursts in cortisol. This may be important for clinicians, in terms of the need to take a continuous series of cortisol measurements from patients rather than a snapshot of their plasma cortisol levels in order to calculate their potential risk of cortisol associated hippocampal dysfunction. We further suggest that it could also be useful to study if the cortisol values which decreased hippocampal output and volume in this simulation mirror ageing-related processes in vivo. The model could also be expanded to include gender differences in chronic stress [22], which may augment neurotoxic effects of elevated plasma cortisol on hippocampal function in females.

Although SBML modelling is in its embryonic stages, skeletal models of physiological systems which share a common computational platform [23], may be useful in the future to test a variety of hypothesis not only in relation to aging but also disease processes. A natural limitation of SBML modelling lies not only in the translation of biophysical interactions into biomathematical equations, but also in the availability of clinical data to use to inform the values of parameters in the model. However, despite these limitations it is possible to generate useful systems models of aging-related neurodegenerative processes, which may be expanded and potentially used by clinicians as prognostic aides.

## Conclusion

We used a variety of Michaelis-Menten and Hill type rate equations to generate a simple steady state model linking resting cortisol levels with hippocampal output. The effects of aging were simulated using a variety of parameters including neuronal loss, a decrease in growth factors, and simulated producing a gradual decrease in HA and HV, at levels not reported to produce cognitive impairment or dementia.

A simulation of the effects of acute increases in plasma cortisol over time produced a decrease in hippocampal activity of 30% and atrophy of 20% by age 90 years. While a chronic elevation of cortisol levels produced a greater decline in hippocampal activity; 40% and greater loss in hippocampal volume 25% – suggesting a chronic elevation in cortisol may be more detrimental in this system rather than an acute elevation. Interestingly the effects of a biological intervention were found to attenuate the effects of chronic cortisol on the hippocampus more greatly than for acute cortisol elevations. Whether or not this in silicointervention is reflecting a real biological process remains to be tested. In theory cognitive impairment associated with a chronic elevation in plasma cortisol may be as a result of structural tissue damage, or transient receptor activation and up-regulation, remodelling of GC associated synapses or simply reflecting a heightened multi-systemic ageing-processes in susceptible older individuals involving a general decrease in health.

We suggest it would be interesting to clinically assess the circulating levels of cortisol at regular intervals in non-cognitively impaired older people who may be at risk of AD. A prospective study should more fully consolidate the link between plasma cortisol levels and cognitive function in older people, in tandem with the use of models such as this, to ascertain if the extrapolated hypothetical effects of increased cortisol level in the elderly are mirrored by clinical changes in cognition. Furthermore, we realise that this paper is framed on the concept that cortisol may induce hippocampal dysfunction. However, hippocampal dysfunction induced by disease processes may uncouple negative feedback by the hippocampus to HPA and alter regulation of cortisol. Hippocampal lesions in Rhesus monkeys alter circulating plasma cortisol levels [3,24]. Therefore it may be interesting to study patients with familial AD, which should not be associated with cortisol as a risk factor, so as to understand if hippocampal related disease processes may be the *a priori *event, which subsequently causes deregulation of cortisol synthesis by the HPA, initiating a self-propelling degenerative process involving ever increasing cortisol levels and increasingly dysfunctional hippocampal neurons.

## Methods

The physiological system modelled is delineated in a simple physiological diagram and flow chart designed to show the relationship between cortisol and hippocampal function (Fig 1a and 1b). SBML was then used to tie together the physiological variables in the model using biochemistry based mathematical questions relating to enzyme substrate reactions (Michaelis-Menten) and receptor ligand interactions (Hill).

The HPA-axis is one of the most studied biological systems and based on available knowledge, a large number of mathematical models of the HPA-axis have been generated [25,29]. Such models have focused on recreating the circadian and ultradian rhythms associated with cortisol secretion over 24 hour oscillations. In this paper we were interested in modelling the effects of elevated cortisol levels on hippocampal function over a forty year period. As people age, while it recognised that the circadian rhythms of cortisol secretion are flattened with altered phase and amplitude in older people [30,31]. In older people with chronic stress disorders, such as post traumatic stress disorders, and in older people with cognitive impairment, cortisol levels are found to be elevated above the normal diurnal or circadian levels [32]. Thus the natural rhythms in cortisol secretion were not focussed on in this first model, rather the impact of age, negative feedback and stress were the main features of the model.

There were two principal model outcomes which we were interested in and which relate to clinically described end points in patients with elevated cortisol levels. The first parameter was hippocampal volume (HV)- indexed to hippocampal atrophy, which was a gross measure of decreased neuronal density and dendritic arborisation. The second parameter as hippocampal output (HO); defined as the combined interaction of CA1 excitatory, inhibitory and aging related signals. The CA1 neuron layer is the major output neuronal subfield in the hippocampus. This parameter does not represent hippocampal output in general; rather a simple measure of its temporal activity particularly in relation to AD and declarative memory.

The full list of model variables is included in Table 1, as are the initial parameter concentrations, and sources used to inform these concentrations, where available. The rate determinants used to define the flow between one variable and another are described in Table 2 and the initial values of the rate constants are detailed in Table 3. The mathematical expressions used to translate the physiological interactions into in silicoequations are further described. In short hippocampal synaptic activity was described using simple input (u) and output (v) terms for the synaptic current (I)[1,33], involving excitatory (Ue) and inhibitory (Ui) synapses and aging related changes in neuronal density, arborisation and growth factors.

**Table 1:** Parameter changes to simulate stress and ageing

Ageing Parameter Changes	Default Value	New Value/Comments
k_hos_	1.6 × 10^-6^	8.1 × 10^-6 ^at 60 years
		5.3 × 10^-5 ^at 80 years
		
k_tfs_	2.5 × 10^-6^	9 × 10^-6 ^at 60 years
		3.4 × 10^-6 ^at 80 years
		
Acute Stress Intervention		
		
Hippocampus Output Chronic Stress Intervention		
k_cor_	2.4 × 10^-3^	Increased default value every year by 5.0 × 10^-5 ^from 60–80 years then reduced parameter by 3.24 × 10^-5 ^to mark cessation of chronic stress
		
Hippocampus output Acute Stress Intervention		
k_cor_	2.4 × 10^-3^	Increased default value every year by 3.0 × 10^-5 ^from 60–80 years then reduced parameter by 2.50 × 10^-5 ^to mark cessation of chronic stress
		
Hippocampus Output Acute Stress Intervention		
k_cor_	2.4 × 10^-3^	Increased default value every year by 1.55 × 10^-5 ^from 60–80 years then reduced parameter by 2.50 × 10^-5 ^to mark cessation of acute stress
		
Hippocampus output chronic stress intervention		
k_cor_	2.4 × 10^-3^	Increased default value every year by 2.05 × 10^-5 ^from 60–80 years then reduced parameter by 1.5 × 10^-5 ^to mark cessation of chronic stress
		
Hippocampus output with PA intervention for acute stress		
k_cor_	2.4 × 10^-3^	Decreased value used for acute stress intervention by 2.8 × 10^-5 ^on 60 years 3.0 × 10^-6^
		
Hippocampus Output with PA intervention for chronic stress		
k_cor_	2.4 × 10^-3^	Decreased value used for acute stress intervention by 1.0 × 10^-5 ^on 60 years and 80 2.5 × 10^-6^
		
Hippocampus Volume With PA intervention for chronic stress		
k_cor_	2.4 × 10^-3^	Decreased value used for acute stress intervention by 5 × 10^-8 ^on 60 years and 80 years 5 × 10^-5^
Hippocampus Volume With PA intervention for acute stress		
k_cor_	2.4 × 10^-3^	Decreased value used for acute stress intervention by 3.0 × 10^-6 ^on 60 years and 80 8.0 × 10^-8^

**Table 2:** Model Species and Initial Values

Abbreviation	Species	Value*
AC	Adrenal cortex	100
ACS	Adrenal cells sink	35 pg/mL (Jacobi, Titze et al. 2001)
ACTH	Adrenocorticotropic hormone	15 μg/dL (Jacobi, Titze et al. 2001)
Cort	Cortisol	35
CRH	Corticotropin-releasing hormone	0
CS	Cortisol sink	100
DGF	Denditic Growth Factors	0
DGFS	Denditic Growth Factors Sink	100
ES	External stress	0
GRA	Active GR receptors	0
GRAS	Active GR receptors sink	1.4 × 10^4 ^(Goncharuk, Van Heerikhuize et al. 2002)
HCS	Hypothalamus secretory cells sink	0
HSC	Hypothalamus secretory cells	100
HTA	Hippocampus Tissue Area	100
HO	Hippocampus output	0
HOS	Hippocampus output sink	0
MRA	Active MR receptors	0
MRAS	Sink for MR active receptors	100
NBD	Neuronal branching; Dendrites	0
NBDS	Neuronal branching dendrites sink	100
NGF	Neuronal Growth factors	100
NGFS	Loss of neuronal growth factors	100
NPOP	Population of neurons in the CA1	4.85 × 10^7 ^(Simic, Kostovic et al. 1997).
NPOPS	Decline in neuronal population of cells	0
PSC	Pituitary secretory cells	1.0*10^7 ^(Trouillas, Guigard et al. 1996)
PCS	Pituitary secretory cell sink	0
Ue	Exitatory synapses	100
Is#	Synaptic current	100
Ui	Inhibitory synapses	0
SISS	Synaptic inhibitory spikes sink	100
V	Synaptic output	100
TF	Trophic Factors	100
TFS	Trophic Factors Sink	0
TM	Tissue mass	70 kg

This table describes the algebraic abbreviations used throughout subsequent mathematical expressions used to describe cortisol synthesis by the HPA and saturation of hippocampal MR and GR receptors with respect to hippocampal activity and aging related atrophy. Values set to 100 are indicative of % activity based on the assumption that the physiological system was healthy at the initiation of the simulation. *Numerical values for neuronal populations etc were extracted from published human clinical studies where available.

**Table 3:** Model Kinetics and Rate Constant Values.

Abbreviation	Description	Default Values
k_crh_	Rate constant for generation of CRH	3.8 × 10^-1^
k_acth_	Rate constant for generation of ACTH	1.085 × 10^-1^
k_cor_	Rate constant for generation of cortisol	2.4 × 10^-3^
k_cdeg_	Rate constant for the degradation of cortisol.	8.5 × 10^-3^
V_crh_	Saturation level of inhibition for hypothalamic cells	1
V_acth_	Saturation level of inhibition for pituitary secretory cells	1
k_da_	Dissociation constant for cortisol in the hypothalamus	50
k_dc_	Dissociation constant for cortisol in the pituitary	50
k_gr_	Concentration of cortisol at which GR receptors are 50% saturated.	1 × 10^8^
k_mr_	Concentration of cortisol at which MR is 50% of its max	2.5 × 10^3^
k_mad_	Rate constant for the deactivation of MR receptors	1
k_non_	Rate constant for the inhibition of CA1 neurons by MR receptors.	4.99 × 10^-2^
k_GRD_	Rate constant for degradation of GR activity	4.45 × 10^-1^
k_ngf_	Rate constant for stimulation of CA1 neurons by growth factors.	10 × 10^-8^
k_nps_	Rate constant for death of neurons.	4.5 × 10^-2^
k_ngfs_	Rate constant for neuronal growth factor decline.	1
k_nbd_	Rate constant for stimulation of NBD	5 × 10^-2^
k_ses_	Rate constant for stimulation of synaptic excitatory signals.	1.049
k_sf_	Rate constant for stimulation of synaptic firing	1.049
k_so_	Rate constant for stimulation of synaptic output.	1.049
k_ho_	Rate constant for stimulation of hippocampal output.	1.049
k_hos_	Rate constant for degradation of synaptic output	5.5 × 10^4^
k_SI_	Rate constant for inhibition of synaptic firing by synaptic inhibitory signals.	2.2 × 10^-1^
k_SGR_	Rate constant for production of synaptic inhibitory signals.	4.45 × 10^-1^
k_sos_	Rate constant for stimulation of synaptic firing.	1.049
k_sisd_	Rate constant for decline in synaptic inhibitory signals	3.8 × 10^-1^
V_crh_	Saturation level of inhibition for hypothalamic cells	1
V_acth_	Saturation level of inhibition for pituitary cells	1
Vmax_MRA_	Maximum rate of activity of MR receptors	1 × 10^2^
Vmax_GRA_	Maximum rate of activity of GR receptors	1 × 10^2^
Ngr	Hill coefficient for GR receptor Activity	3.6
Nmr	Hill coefficient for MR receptor activity	5.8
HV	Volume of Hippocampus	100

This table describes the rate constant expressions used to define the interaction between variables within the model. Rate constants were extrapolated and simplified from known biophysical and biochemical reactions within hippocampal and HPA tissues. Michalelis-Menton steady state enzyme substrate reactions and the Hill receptor-ligand binding equations were used to define interactions between model variables.

### 1 HPA Regulation of Cortisol Secretion

The secretion of cortisol via HPA regulation was assumed to be in a "steady-state" and represented using a standard "Michaelis-Menten" type biochemical equations and rate equations (Eq 1–3).

(1)

(2)

(3)

### 2 Cortisol's Interaction with MR and GR Receptors

The binding of cortisol to CA1 MR receptors was represented using the Hill equation for ligand/receptor binding. The first half of the ordinary differential equation (ODE) represents the generation of MR activity, while the second half of the ODE is a degradation reaction, which is necessary to ensure that MR activity does not rise indefinitely (Eq 4).

(4)

The binding of cortisol to GR CA1 hippocampal neurons was expressed also using the Hill equation, and combined with a degradation reaction similar to Eq 4, to ensure that GR receptor activity does not rise indefinitely (Eq 5).

(5)

### 3 Aging of CA1 neurons and cortisol stimulation

The population of neurons in the CA1 region of the hippocampus was defined by combining the stimulation of the neuronal population by neuronal growth factors and MR activity, with the last part of the equation representing the ageing related death of neurons (Eq 6).

(6)

A rate constant for the decline of neuronal growth factors with time was also included (Eq 7).

(7)

### 4 Excitatory Input Synapses

Exitatory input synapses (Ue) were defined with reference to the relationship between the neuronal population and excitatory impulses with degradation of neuronal arborisation (Eq 8).

(8)

It was assumed that the numbers of excitatory synapses would be related both to the numbers of neuronal branches (dendrites) and also synaptic excitatory signals (Eq 9).

(9)

### 5 Inhibitory Input Synapses

Inhibitory synapses (Ui) were defined by combining the activation of GR receptors with the degradation of synaptic inhibitory signals (Eq 10).

(10)

### 6 Aging related changes in Synaptic Current [Is]

Synaptic current was defined as the net combination of excitatory and inhibitory input synapses (Eq 11).

(11)

### 7 Synaptic Output

Synaptic output (Vs) was defined as a combination of synaptic output with a decline of synaptic output related to time (Eq 12).

(12)

### 8 Hippocampal Atrophy

In the model hippocampus atrophy was defined using an SBML rule which was implemented in MathSBML as detailed below.

(13)

### 9 Hippocampal Tissue Output

The first half of the equation represented the generation of hippocampus output (HO), while the second half of the equation represented the degradation of hippocampus output. The degradation of HO had no biological significance, but served to ensure that hippocampus output did not rise indefinitely when simulations were conducted but instead reached a steady state (Eq 14).

(14)

Firstly the system model was brought into a steady state, and the initial hippocampus output was set at 100% which is a mathematical representation of activity, and does not refer to the cognitive ability of the in silicoindividual modelled in this paper.

The response to stress was examined by using events in SBML designed to mirror physiological responses to stress. The first event triggered an increase in the reaction rate k_crh_, which raised the secretion of CRH. This in turn precipitated an increase in ACTH, followed by an increase in plasma cortisol. The second event returned k_crh _to its original value after a short period of time which produced a yearly increase in cortisol. k_cr _was not returned to its original value after each event in order so as to represent stress and ageing altering the ability of the HPA-axis to recover from repeated challenges which reflects the clinical hypothesis that ageing impairs homeostatic adaptations of cortisol secretion to stress.(31).

## Authors' contributions

MMA was responsible for the majority of the computational mathematical modeling and sensitivity analysis of the system. VM was responsible for the initial physiological modeling, data mining and assisted the translation of this model to a mathematical based structure. RAK provided clinical information for the model, and TK provided insight into the effects of aging on the system, while DW and JJ advised on the modelling of the system.

All authors read and approved the final manuscript.

## Appendix

### Necessary changes to ODEs to produce a diurnal cortisol rhythm

(15)

(16)

(17)

k_1 _= 20, k_2 _= 100, k_3 _= 1.05, k_4 _= 1.1, k_5 _= 0.15, k_6 _= 0.6, k_7 _= 10
